# Quadriceps muscle strength, radiographic knee osteoarthritis and knee pain: the ROAD study

**DOI:** 10.1186/s12891-015-0737-5

**Published:** 2015-10-16

**Authors:** Shigeyuki Muraki, Toru Akune, Masatoshi Teraguchi, Ryohei Kagotani, Yoshiki Asai, Munehito Yoshida, Fumiaki Tokimura, Sakae Tanaka, Hiroyuki Oka, Hiroshi Kawaguchi, Kozo Nakamura, Noriko Yoshimura

**Affiliations:** Department of Clinical Motor System Medicine, 22nd Century Medical & Research Center, Faculty of Medicine, the University of Tokyo, Hongo 7-3-1, Bunkyo-ku, Tokyo 113-8655 Japan; National Rehabilitation Center for Persons with Disabilities, Saitama, Japan; Department of Orthopaedic Surgery, Wakayama Medical University, Wakayama, Japan; Department of Orthopaedic Surgery, Tokyo Geriatric Medical Center, Tokyo, Japan; Department of Orthopaedic Surgery, Faculty of Medicine, the University of Tokyo, Tokyo, Japan; Department of Medical Research and Management for Musculoskeletal Pain, 22nd Century Medical & Research Center, Faculty of Medicine, the University of Tokyo, Tokyo, Japan; Department of Orthopaedic Surgery, Japan Community Health care Organization Tokyo Shinjuku Medical Center, Tokyo, Japan; Department of Joint Disease Research, 22nd Century Medical & Research Center, Faculty of Medicine, the University of Tokyo, Tokyo, Japan

**Keywords:** Cohort study, Epidemiology, Osteoarthritis, Pain, Muscle

## Abstract

**Background:**

The objective of this study was to clarify the association of quadriceps muscle strength with knee pain using a large-scale, population-based cohort of the Research on Osteoarthritis/osteoporosis Against Disability (ROAD) study.

**Methods:**

From the 2566 subjects at the third visit of the ROAD study, the present study analyzed 2152 subjects who completed radiographic examinations and measurements of muscle strength and mass (690 men and 1462 women; mean age, 71.6 ± 12.2 years). Knee pain was assessed by an experienced orthopedist. Knee osteoarthritis (OA) was defined according to Kellgren-Lawrence (KL) grade. Quadriceps muscle strength and muscle mass at the lower limbs were measured by the Quadriceps Training Machine (QTM-05F, Alcare Co., Ltd. Tokyo, Japan) and the Body Composition Analyzer MC-190 (Tanita Corp., Tokyo, Japan), respectively.

**Results:**

Quadriceps muscle strength and weight bearing index (WBI: quadriceps muscle strength by weight) were significantly associated with knee pain after adjustment for age and body mass index, whereas grip strength and muscle mass at the lower limbs were not. The significant association of quadriceps muscle strength with knee pain was independent of radiographic knee OA.

**Conclusion:**

The present cross-sectional study showed an independent association of quadriceps muscle strength with knee pain.

**Electronic supplementary material:**

The online version of this article (doi:10.1186/s12891-015-0737-5) contains supplementary material, which is available to authorized users.

## Background

Knee osteoarthritis (OA) is a major public health issue that causes chronic pain and disability [1–3]. The prevalence of radiographic knee OA is high in Japan [4], with 25,300,000 persons aged 40 and older estimated to have radiographic knee OA [5]. According to the recent National Livelihood Survey of the Ministry of Health, Labour and Welfare in Japan, OA is ranked fourth among diseases that cause disabilities that subsequently require support with activities of daily living [6]. The principal clinical symptom of knee OA is knee pain [7]. Although much effort has been devoted toward a definition of knee pain, its correlation with radiographic severity of knee OA is not as strong as one would expect [4, 8–10]. In fact, our previous study showed that the odds ratio (OR) of severe knee OA defined as Kellgren-Lawrence (KL) grade 3 or 4 for knee pain was 8.6 in men and 4.4 in women [4], which was significant, but the OR was not as high as expected. In addition, 10 % of men without radiographic knee OA and 20 % of women without radiographic knee OA had knee pain [4]. This indicates that at least 10 % and 20 % of knee pain in men and women, respectively, may be explained by factors other than radiographic changes.

One of the factors contributing to knee pain other than radiographic knee OA may be quadriceps muscle weakness. Thus far, grip strength has been used as a useful clinical marker of sarcopenia [11], because measuring grip strength is easy. Although there is growing evidence that reduced grip strength is associated with adverse outcomes including morbidity [12], disability [13], falls [13], higher fracture rates [14], increased length of hospital stay [15], quality of life [16] and mortality [13], and grip strength is related to total muscle strength [17], quadriceps muscle strength may be more strongly associated with knee symptoms than grip strength. However, to the best of our knowledge, no population-based study has compared the association of knee pain with grip strength and quadriceps muscle strength because isokinetic devices such as Cybex, Biodex, and KIN-COM, which allow for the most detailed measurements regarding the quantitative evaluation of the quadriceps muscle strength, are expensive, large-scale, and impossible to move. Recently, the Quadriceps Training Machine (QTM) (QTM-05F, Alcare Co., Ltd. Tokyo, Japan) was developed to measure quadriceps muscle strength more easily [18]. The QTM has higher usability compared with other devices in terms of its small size, light weight, and good portability, as well as the fact that it has good correlation with Biodex and high credibility of measurements [18]. Although measurements of muscle mass are another method to evaluate muscle, the association between muscle strength and mass has been shown to be weak [19], indicating that a distinct association with knee symptoms between quadriceps muscle strength and muscle mass at the lower limb may be found. However, there are no population-based studies that compare the association of knee pain with quadriceps muscle strength and muscle mass at the lower limbs.

The objective of this study was to clarify the association of quadriceps muscle strength and muscle mass at the lower limbs with pain at the knee among Japanese men and women in a large-scale, population-based cohort from the Research on Osteoarthritis/osteoporosis Against Disability (ROAD) study.

## Methods

### Subjects

The ROAD study is a nationwide prospective study designed to establish epidemiologic indices for the evaluation of clinical evidence for the development of a disease-modifying treatment for bone and joint diseases (with OA and osteoporosis as the representative bone and joint diseases). It consists of population-based cohorts in several communities in Japan. A detailed profile of the ROAD study has been reported elsewhere [4, 5, 20], and thus, only a brief summary is provided here. To date, we have completed the creation of a baseline database including clinical and genetic information for 3040 inhabitants (1061 men and 1979 women) ranging in age from 23 to 95 years (mean, 70.3 years), who were recruited from resident registration listings in three communities: an urban region in Itabashi, Tokyo, a mountainous region in Hidakagawa, Wakayama, and a coastal region in Taiji, Wakayama. All participants provided written, informed consent, and the study was conducted with the approval of the ethics committees of the University of Tokyo and the Tokyo Metropolitan Institute of Gerontology.

The third visit of the ROAD study began in 2011 and was completed in 2013. All participants in the baseline study were invited to participate in the third visit. In addition to the former participants, inhabitants aged ≥60 years in the urban area and those aged ≥40 years in the mountainous and coastal areas who were willing to participate in the ROAD survey performed in 2011–2013 were also included in the third visit.

Anthropometric measurements, including height and weight, were taken, and body mass index (BMI; weight [kg]/height^2^ [m^2^]) was calculated. Grip strength was measured on the right and left sides using a TOEI LIGHT handgrip dynamometer (TOEI LIGHTCO. LTD, Saitama, Japan). Isometric quadriceps muscle strength at the right and left knee was measured by the QTM one time each, and weight bearing index (WBI: quadriceps muscle strength/body weight) was calculated. Subjects carried out knee extension exercises by placing their knee joint on the QTM where specified; the load pressure applied to the QTM in the popliteal region was measured and displayed as the isometric knee extension muscle strength (quadriceps strength). The QTM has good correlation with Biodex and high credibility of measurement, and the method has been validated [18]. Lower limb muscle mass was measured by bioimpedance analysis [21–24] using the Body Composition Analyzer MC-190 (Tanita Corp., Tokyo, Japan), and muscle mass/height^2^ (kg/m^2^) was calculated. The protocol was described by Tanimoto and colleagues [25, 26], and the method has been validated [27].

All participants were also interviewed by well-experienced orthopedists regarding pain in both knees, by asking: “Have you experienced right knee pain on most days in the past month, in addition to now?” and “Have you experienced left knee pain on most days in the past month, in addition to now?”. Subjects who answered “yes” were defined as having knee pain.

### Radiographic assessment

All participants underwent radiographic examination of both knees using an anterior-posterior view with weight-bearing and foot map positioning by experienced radiological technologists. The beam was positioned parallel to the floor with no angle and aimed at the joint space. To visualize the joint space properly and to centralize the patella over the lower end of the femur, fluoroscopic guidance with an anterior-posterior X-ray beam was used, and the images were downloaded into Digital Imaging and Communication in Medicine (DICOM) format files. Knee radiographs were read without knowledge of participant clinical status by a single experienced orthopedist (S.M.) using the KL radiographic atlas for overall knee radiographic grades [28], and knee OA was defined as KL grade 2 or greater. To evaluate the intraobserver variability of the KL grading, 100 randomly selected radiographs of the knee were scored by the same observer more than 1 month after the first reading. One hundred other radiographs were also scored by two experienced orthopedic surgeons (S.M. & H.O.) using the same atlas for interobserver variability. The intra- and inter-variabilities evaluated for KL grade (0-4) were confirmed by kappa analysis to be sufficient for assessment (0.86 and 0.80, respectively).

### Statistical analysis

Differences in age, height, weight, BMI, muscle strength, WBI and muscle mass between men and women and between subjects with and without pain were examined using the non-paired student t-test. The prevalence of knee OA and pain was compared between men and women by the χ^2^ test. Linear regression analysis was used to determine the association of age, muscle mass at the lower limb, and grip strength with quadriceps muscle strength. Associations of age, BMI, grip strength, quadriceps muscle strength, WBI and muscle mass at the lower limbs and KL grade with knee pain were determined using multiple logistic regression analysis after adjustment for age, sex, and BMI overall, and after adjustment for age and BMI in men and women. To determine the independent association of age, BMI, gender, muscle strength, and KL grade with knee pain, multiple logistic regression analysis was used with age, BMI, gender, muscle strength, and KL grade overall, and with age, BMI, muscle strength, and KL grade in men and women, as explanatory variables. To determine the independent association of WBI with knee pain, multiple logistic regression analysis was used with age, BMI, gender, WBI and KL grade, overall, and with age, BMI, WBI and KL grade in men and women as explanatory variables. In addition, subjects were classified according to muscle strength (<10 kgf, ≥10– < 20 kgf, ≥20– < 30 kgf, ≥30– < 40 kgf, ≥40 kgf), and the association of muscle strength <10 kgf, ≥10– < 20 kgf, ≥20– < 30 kgf, and ≥30– < 40 kgf with pain was determined using multiple logistic regression analysis after adjustment for age and BMI, compared with muscle strength ≥40 kgf). The thresholds of muscle strength or WBI for pain were determined using ROC curve analysis. Data analyses were performed using SAS version 9.0 (SAS Institute Inc., Cary, NC).

## Results

Among the 2566 subjects who participated in the third visit of the ROAD study, 2303 (89.9 %) subjects underwent X-ray examinations at the knee. A total of 32 (1.3 %) subjects who underwent total knee arthroplasty before the third visit were excluded from the study. In addition, 12 (0.5 %) subjects who provided incomplete questionnaires regarding pain and 37 subjects (1.5 %) who did not undergo an examination of muscle strength or muscle mass were excluded. Further, 58 subjects (2.3 %) who were younger than 40 years were excluded, leaving a total of 2152 (85.1 %) subjects (690 men and 1462 women). The characteristics of the 2152 participants in the present study are shown in Table 1. Muscle strength and mass were significantly higher in men than women. WBI was not significantly different between men and women. The prevalence of knee OA and knee pain was significantly higher in women than in men. Quadriceps muscle strength was significantly associated with muscle mass at the lower limbs, but the association was weak (right: correlation coefficient =0.28 and 0.21 in men and women, respectively, *p* < 00001; left: correlation coefficient 0.34 and 0.37 in men and women, respectively, *p* < 00001). Quadriceps muscle strength was also significantly associated with grip strength, and the association was moderate (right: correlation coefficient =0.47 and 0.50 in men and women, respectively, *p* < 00001; left: correlation coefficient 0.50 and 0.52 in men and women, respectively, *p* < 00001). Quadriceps muscle strength was significantly associated with age in men and women (*p* < 0.0001) (Additional file 1: Figure S1).

**Table 1:** Subject characteristics

	Overall	Men	Women	*P* values
N	2152	690	1462	
Age, years	71.6 ± 12.2	72.5 ± 12.3	71.2 ± 12.1	0.0164
Height, cm	154.3 ± 9.2	163.1 ± 7.1	150.1 ± 6.8	<0.0001
Weight, kg	54.3 ± 10.7	61.6 ± 11.0	50.9 ± 8.6	<0.0001
BMI, kg/m^2^	22.7 ± 3.4	23.1 ± 3.3	22.5 ± 3.5	0.0009
*Right*				
Grip strength	28.1 ± 9.6	37.2 ± 9.4	23.7 ± 5.8	<0.0001
Quadriceps muscle strength, kgf	28.1 ± 11.2	31.9 ± 12.7	26.2 ± 10.0	<0.0001
Weight bearing index	0.52 ± 0.20	0.52 ± 0.20	0.52 ± 0.20	0.8724
Lower limb muscle mass, kg	6.3 ± 1.6	7.9 ± 1.5	5.5 ± 0.8	<0.0001
Lower limb muscle mass/height^2^, kg/m^2^	2.6 ± 0.4	3.0 ± 0.4	2.4 ± 0.3	<0.0001
Knee OA (%)	44.1	31	50.3	<0.0001
Knee pain (%)	20.6	15.1	23.3	<0.0001
*Left*				
Grip strength	26.2 ± 9.4	35.2 ± 9.1	22.0 ± 5.9	<0.0001
Quadriceps muscle strength, kgf	26.9 ± 11.2	30.6 ± 12.6	25.1 ± 9.9	<0.0001
Weight bearing index	0.50 ± 0.20	0.50 ± 0.20	0.50 ± 0.20	0.9715
Lower limb muscle mass, kg	6.2 ± 1.6	7.8 ± 1.5	5.4 ± 0.8	<0.0001
Lower limb muscle mass/height^2^, kg/m^2^	2.6 ± 0.4	2.9 ± 0.4	2.4 ± 0.3	<0.0001
Knee OA (%)	45.2	33	51	<0.0001
Knee pain (%)	20	13.9	22.9	<0.0001

Except where indicated otherwise, values are means ± SD

Knee OA was defined as Kellgren-Lawrence grade 2 or worse

Weight bearing index was calculated as quadriceps muscle strength by weight

Differences between men and women were determined by non-paired student t test except for prevalence of knee OA and knee pain

Differences in prevalence of knee OA and knee pain between men and women were determined by chi-square test

*BMI* Body mass index, *OA* Osteoarthritis

Table 2 shows age, BMI, grip strength, quadriceps muscle strength, WBI and lower limb muscle mass/height^2^ in subjects with and without pain. For the right knee, age, BMI, grip strength, quadriceps muscle strength and WBI were significantly different between subjects with and without pain, whereas muscle mass was not. Results were similar for the left knee. After adjustment for age and BMI, the significant association of grip strength with knee pain disappeared in men and women.

**Table 2:** Age, BMI, grip strength and lower limb muscle strength and muscle mass in subjects with and without knee pain

	Right knee					Left knee				
	Pain -	Pain +	Adjusted OR	95 % CI	*P* values	Pain -	Pain +	Adjusted OR	95 % CI	*P* values
Overall										
N	1708	444				1721	431			
Age, years	70.8 ± 12.5	74.8 ± 10.4*	1.04	1.03–1.035	<0.0001	70.8 ± 12.5	74.7 ± 10.4*	1.04	1.03–1.05	<0.0001
BMI, kg/m^2^	22.5 ± 3.3	23.7 ± 3.7*	1.13	1.10–1.17	<0.0001	22.4 ± 3.3	23.8 ± 3.5*	1.14	1.11–1.18	<0.0001
Grip strength, kgf	28.7 ± 9.7	25.5 ± 8.5*	0.98	0.96–0.9996	0.0448	26.9 ± 9.5	23.8 ± 8.5*	0.99	0.97–1.01	0.3464
Quadriceps muscle strength,kgf (5kgf increase)	29.1 ± 11.2	23.9 ± 10.3*	0.83	0.78–0.88	<0.0001	27.9 ± 11.2	22.8 ± 10.2*	0.84	0.79–0.89	<0.0001
Weight bearing index, kgf/kg (0.1 kgf/kg increase)	0.54 ± 0.20	0.44 ± 0.18*	0.81	0.76–0.86	<0.0001	0.52 ± 0.19	0.42 ± 0.19*	0.83	0.77–0.88	<0.0001
Lower limb muscle mass/height^2^,kg/m^2^ (0.1kg/m^2^ increase)	2.59 ± 0.43	2.58 ± 0.43	0.97	0.92–1.02	0.2421	2.56 ± 0.43	2.56 ± 0.42	0.98	0.94–1.03	0.4326
Men										
N	586	104				594	96			
Age, years	71.9 ± 12.5	76.1 ± 11.0*	1.04	1.02–1.07	<0.0001	72.0 ± 12.3	76.1 ± 11.7*	1.04	1.02–1.07	<0.0001
BMI, kg/m^2^	22.9 ± 3.2	23.9 ± 3.8*	1.13	1.06–1.21	0.0002	22.9 ± 3.2	23.9 ± 4.0*	1.13	1.06–1.21	0.0003
Grip strength, kgf	37.5 ± 9.5	35.1 ± 9.0*	0.98	0.95–1.01	0.3070	35.5 ± 9.0	33.3 ± 9.7*	0.99	0.96–1.02	0.5031
Quadriceps muscle strength,kgf (5kgf increase)	32.9 ± 12.5	26.4 ± 12.0*	0.80	0.72–0.89	<0.0001	31.4 ± 12.6	25.4 ± 11.4*	0.82	0.91–1.23	0.0001
Weight bearing index, kgf/kg (0.1 kgf/kg increase)	0.54 ± 0.20	0.42 ± 0.19*	0.75	0.65–0.85	<0.0001	0.52 ± 0.20	0.41 ± 0.18*	0.78	0.68–0.89	0.0002
Lower limb muscle mass/height^2^, kg/m^2^ (0.1kg/m^2^ increase)	2.95 ± 0.44	3.03 ± 0.47	1.01	0.92–1.10	0.8897	2.90 ± 0.44	2.97 ± 0.50	0.98	0.89–1.08	0.7281
Women										
N	1122	340				1127	335			
Age, years	70.2 ± 12.5	74.4 ± 10.2*	1.03	1.02–1.05	<0.0001	70.2 ± 12.6	74.3 ± 10.0*	1.03	1.02–1.05	<0.0001
BMI, kg/m^2^	22.2 ± 3.3	23.7 ± 3.6*	1.13	1.09–1.18	<0.0001	22.2 ± 3.4	23.8 ± 3.4*	1.15	1.11–1.19	<0.0001
Grip strength, kgf	24.1 ± 5.8	22.5 ± 5.8*	0.98	0.95–1.004	0.1014	22.3 ± 6.0	21.1 ± 5.7*	0.99	0.97–1.02	0.6256
Quadriceps muscle strength, kgf (5kgf increase)	27.2 ± 9.9	23.2 ± 9.5*	0.84	0.78–0.91	<0.0001	26.1 ± 9.8	22.1 ± 9.7*	0.85	0.79–0.91	<0.0001
Weight bearing index, kgf/kg (0.1 kgf/kg increase)	0.55 ± 0.20	0.45 ± 0.18*	0.83	0.77–0.90	<0.0001	0.52 ± 0.19	0.43 ± 0.19*	0.84	0.78–0.91	<0.0001
Lower limb muscle mass/height^2^,kg/m^2^ (0.1kg/m^2^ increase)	2.41 ± 0.27	2.45 ± 0.32*	0.95	0.89–1.02	0.1421	2.38 ± 0.29	2.44 ± 0.31*	0.99	0.93–1.04	0.6166

*p < 0.05 by non-paired student t test

Adjusted ORs were calculated by multiple logistic regression analysis after adjustment for age, sex, and BMI overall and after adjustment for age and BMI in men and women

*BMI* Body mass index, *mJSW*, Minimum joint space width

**Fig. 1:**
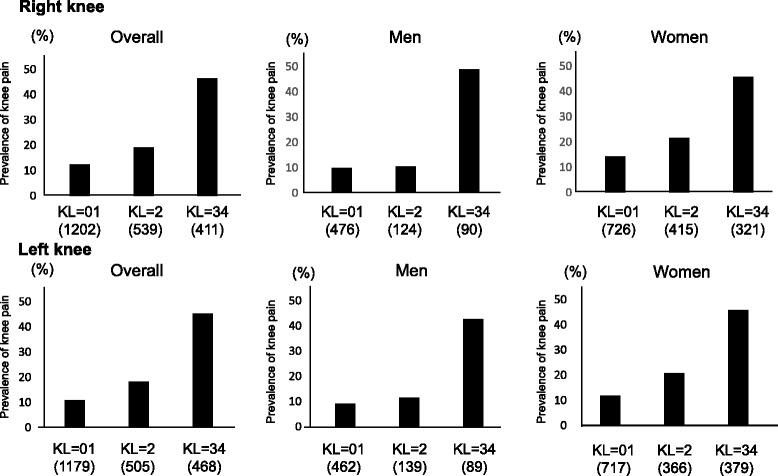
Prevalence of knee pain by Kellgren-Lawrence (KL) grade. The number of subjects in each KL grade is shown in parentheses

We next examined the prevalence of knee pain according to KL grade (Fig. 1). In the overall population, the prevalence of knee pain was 12.5 %, 19.1 % and 46.5 % in the right knee and 10.8 % 18.2 % and 45.3 % in the left knee in subjects with KL = 01, KL = 2 and KL = 3 or 4, respectively. After adjustment for age, gender and BMI, KL = 3 or 4 was significantly associated with knee pain compared with KL = 01 (right knee: odds ratio [OR] 4.16, 95 % confidence interval [CI] 3.10-5.61; left knee: OR 4.90, 95 CI 3.63-6.64). KL = 2 at the left knee was also significantly associated with pain (OR 1.52, 95 % CI 1.17-2.00), while KL = 2 at the right knee was not (OR 1.27, 95 % CI 0.94-1.71). The prevalence of knee pain was 9.9 %, 10.5 % and 48.9 % at the right knee and 9.1 %, 11.5 and 42.7 % at the left knee in men with KL = 01, KL = 2 and KL = 3 or 4, respectively, and 14.2 %, 21.7 % and 45.8 % at the right knee and 11.9 %, 20.8 % and 45.9 % at the left knee in women with KL = 01, KL = 2 and KL = 3or 4, respectively. In men and women, after adjustment for age and BMI, KL = 3 or 4 was significantly associated with knee pain at the right knee (men: OR 6.82, 95 % CI 3.94-11.9, women: OR 3.52, 95 % CI 2.49-5.03) and the left knee (men: OR 5.64, 95 % CI 3.20-9.99, women: OR 4.83, 95 % CI 3.39-6.94). KL = 2 was not associated with knee pain except for the left knee in women (right knee, men: OR 0.91, 95 % CI 0.45-1.73, women: 1.32, 95 % CI 0.93-1.86; left knee, men: OR 1.08, 95 % CI 0.56-2.00, women: 1.68, 95 % CI 1.16-2.45).

To determine independent associations of age, BMI, gender, muscle strength and knee OA, we next used multiple logistic regression analysis with age, BMI, gender, quadriceps muscle strength and KL grade as explanatory variables in subjects overall, and with age, BMI, muscle strength and KL grade as explanatory variables in men and women (Table 3). Overall, BMI, gender, muscle strength and KL grade 3 or 4 were significantly associated with knee pain, whereas age and KL grade 2 were not. In men and women, BMI, muscle strength and KL grade 3 or 4 were significantly associated with knee pain. We also analyzed independent associations of age, BMI, gender, WBI and knee OA. Results for WBI were almost the same as those for quadriceps muscle strength (overall: OR 0.85, 95 % CI 0.79-0.91, *p* = 0.0011, men: OR 0.79, 95 % CI 0.69-0.90, *p* = 0.0003, women: OR 0.87, 95 % CI 0.80-0.94, *p* = 0.0003).

**Table 3:** Association of age, BMI, gender, muscle strength and severity of knee OA with knee pain

	Right knee			Left knee		
	Adjusted OR	95 % CI	*P* values	Adjusted OR	95 % CI	*P* values
Overall						
Age	1.01	0.996–1.02	0.1698	1.00	0.99–1.02	0.6107
BMI	1.09	1.06–1.13	<0.0001	1.09	1.06–1.13	<0.0001
Women (vs Men)	1.34	1.03–1.76	0.0299	1.35	1.03–1.78	0.0321
Quadriceps muscle strength (5kgf increase)	0.87	0.82–0.92	<0.0001	0.88	0.82–0.93	<0.0001
KL 2	1.3	0.96–1.75	0.0929	1.54	1.12–2.12	0.0083
KL 3 or 4	3.77	2.79–5.10	<0.0001	4.49	3.31–6.10	<0.0001
Men						
Age	1.01	0.99–1.04	0.3309	1.01	0.99–1.04	0.3269
BMI	1.09	1.02–1.17	0.013	1.09	1.02–1.18	0.0152
Quadriceps muscle strength (5 kgf increase)	0.85	0.76–0.94	0.0019	0.86	0.77–0.96	0.0087
KL 2	1.06	0.54–2.16	0.8753	1.18	0.60–2.21	0.624
KL 3 or 4	5.98	3.42–10.54	<0.0001	4.99	2.79–8.93	<0.0001
Women						
Age	1.01	0.99–1.02	0.2416	1.00	9.99–1.02	0.945
BMI	1.09	1.05–1.14	<0.0001	1.09	1.05–1.14	<0.0001
Quadriceps muscle strength (5 kgf increase)	0.88	0.82–0.95	0.0007	0.89	0.82–0.96	0.003
KL 2	1.33	0.95–1.89	0.1007	1.67	1.15–2.44	0.0068
KL 3 or 4	3.24	2.28–4.64	<0.0001	3.37	3.12–6.44	<0.0001

Adjusted OR was calculated by multiple logistic regression analysis with age, BMI, gender, Quadriceps muscle strength and KL grade as explanatory variables

*OR* Odds ratio, *CI* Confidence interval, *BMI*, Body mass index

**Fig. 2:**
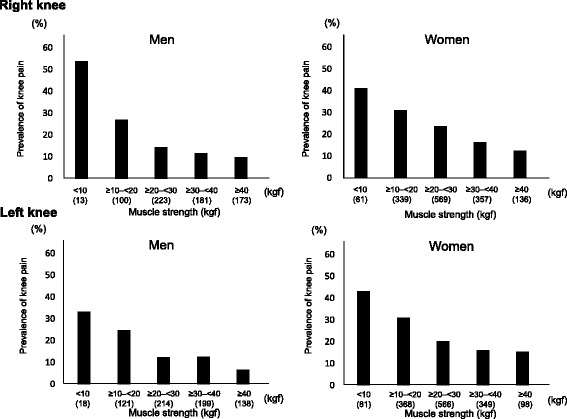
Prevalence of knee pain by muscle strength at the lower limb in men and women. The number of subjects in each muscle strength is shown in parentheses

**Fig. 3:**
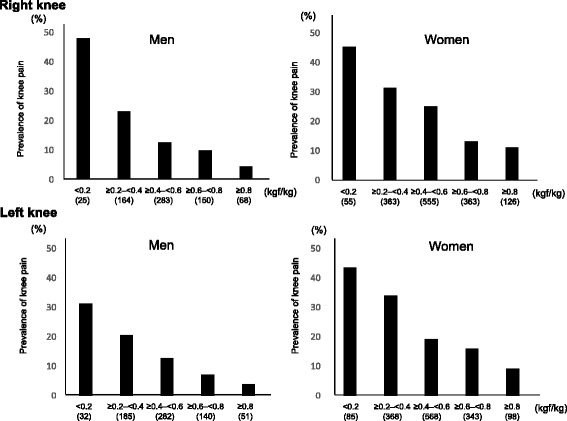
Prevalence of knee pain by weight bearing index in men and women. The number of subjects in each muscle strength is shown in parentheses. Weight bearing index was calculated as quadiceps muscle strength/weight

Next, to determine the prevalence of knee pain according to muscle strength, subjects were classified by muscle strength (<10 kgf, ≥10– < 20 kgf, ≥20– < 30 kgf, ≥30– < 40 kgf, ≥40 kgf). Prevalence of knee pain was 53.9 %, 27.0 %, 14.4 %, 11.6 and 9.8 % at the right knee and 33.3 %, 24.8 %, 12.2 %, 12.6 % and 6.5 % at the left knee in men with muscle strength <10 kgf, ≥10– < 20 kgf, ≥20– < 30 kgf, ≥30– < 40 kgf and ≥40 kgf, respectively, and 41.0 %, 31.0 %, 23.7 %, 16.3 % and 12.5 % at the right knee and 43.2 %, 31.0 %, 20.3 %, 16.1 % and 15.3 % at the left knee in women with muscle strength <10 kgf, ≥10– < 20 kgf, ≥20– < 30 kgf, ≥30– < 40 kgf and ≥40 kgf, respectively (Fig. 2). After adjustment for age, BMI and KL grade, subjects with muscle strength <10 kgf and ≥10– < 20 kgf had a significantly higher prevalence of knee pain compared with those with muscle strength ≥40kgf, except for left knee in women (Table 4). We also examined the prevalence of knee pain according to WBI and found similar results (Fig. 3).

**Table 4:** Odds ratio for knee pain based on quadriceps muscle strength

	Right knee			Left knee		
	OR	95 % CI	*P* value	OR	95 % CI	*P* value
Men						
< 10 kgf	5.87	1.46–23.5	0.0131	4.00	1.002–15.4	0.0497
≥ 10- < 20 kgf	2.26	1.08–4.83	0.0312	3.03	1.30–7.59	0.0096
≥ 20- < 30 kgf	0.95	0.48–1.92	0.8909	1.39	0.61–3.44	0.4405
≥ 30- < 40 kgf	1.14	0.56–2.32	0.7230	1.74	0.79–4.16	0.1771
≥ 40 kgf	1			1		
Women						
< 10 kgf	2.78	1.28–6.13	0.0095	2.00	0.93–4.45	0.0783
≥ 10- < 20 kgf	1.82	1.01–3.42	0.0452	1.49	0.79–2.94	0.2253
≥ 20- < 30 kgf	1.7	0.98–3.10	0.0612	1.03	0.56–2.00	0.9227
≥ 30- < 40 kgf	1.11	0.62–2.08	0.7274	0.91	0.48–1.80	0.7879
≥ 40 kgf	1			1		

*OR* Odds ratio, *CI* Confidence interval

The threshold values of muscle strength for knee pain were then determined using ROC curve analysis. At the right knee, the threshold values of muscle strength for pain were 27.5 kgf (sensitivity 0.58, specificity 0.64, AUC 0.64) and 27.0 kgf (sensitivity 0.72, specificity 0.48, AUC 0.62) in men and women, respectively. At the left knee, the threshold values of muscle strength for pain were 20 kgf (sensitivity 0.39, specificity 0.82, AUC 0.64) and 23.2 kgf (sensitivity 0.59, specificity 0.41, AUC 0.61) in men and women, respectively. Regarding WBI, the threshold values for pain were 0.43 kgf/kg (sensitivity 0.57, specificity 0.69, AUC 0.67) in men and 0.49 kgf/kg (sensitivity 0.64, specificity 0.59, AUC 0.64) in women at the right knee, and 0.37 kgf/kg (sensitivity 0.46, specificity 0.78, AUC 0.65) in men and 0.40 kgf/kg (sensitivity 0.49, specificity 0.74, AUC 0.64) in women at the left knee.

## Discussion

This is the first study to clarify the effect of quadriceps muscle strength as well as muscle mass on knee pain using a large-scale, population-based, cohort study. In the present study, quadriceps muscle strength was significantly associated with knee pain, while grip strength and muscle mass of the lower limb were not. The significant association of quadriceps muscle strength with knee pain remained after adjustment for age, BMI, gender and knee OA.

The present study first clarified that quadriceps muscle strength and WBI were significantly associated with knee pain even after adjustment for radiographic knee OA, which means that the association of muscle strength with knee pain is independent of radiographic changes. In fact, our previous and other previous studies had already shown that the correlation with radiographic severity of the knee OA was not as strong as one would expect [4, 8–10], indicating that there may be some factors other than radiographical changes to explain knee pain. Our results in the present study indicate that not only radiographical changes but also quadriceps muscle strength has an important role in knee pain. The quadriceps muscle is the principal dynamic stabilizer of the knee joint; thus, quadriceps muscle weakness leads to instability of the knee, which may be one of the reasons for knee pain. This also means that knee pain may be prevented by muscle exercise. However, around 10 % of subjects with ≥40 kgf muscle strength had knee pain, indicating that several other factors such as synovitis, knee alignment, meniscal degeneration, thrust and so on may also affect knee pain.

In the present study, although the association of quadriceps muscle strength and grip strength was moderate, quadriceps muscle strength rather than grip strength was significantly associated with knee pain. The QTM used in the present study has higher usability compared with other devices. Thus, to use not only grip strength but also quadriceps muscle strength by the QTM may be recommended to estimate sarcopenia.

In the present study, we also examined muscle mass in the lower limbs and found that the association of muscle mass with quadriceps muscle strength was weak. This may be partly explained by impaired neuromuscular activation, which has an independent contribution to muscle strength after adjustment for muscle mass [29]. Furthermore, several studies reported that greater thigh adiposity is known to be associated with lower strength, worse mobility, and worse lipoprotein profiles in the elderly [30–32], which may obscure the association between muscle strength and mass at the lower limbs. This also may be partly explained by the fact that we examined muscle mass not at the quadriceps but at the whole limb on the right and left sides, because the Body Composition Analyzer MC-190 used in the present study cannot measure only quadriceps muscle mass. The present study also showed that muscle strength rather than muscle mass at the lower limbs was associated with knee pain. Previous studies found that lower limb muscle strength, but not muscle mass, was associated with quality of life [19]. Greater thigh adiposity and impaired neuromuscular function may also obscure the association of muscle mass with knee pain.

In the present study, sex differences were found in the association of quadriceps muscle strength with pain. The OR of muscle strength <10 kgf for pain was approximately 5 in men compared with muscle strength ≥40 kgf, while it was approximately 2 in women. These discrepancies between the sexes are partly explained by the fact that women are more susceptible to pain than men [4]. In fact, our previous study showed that the OR for knee pain in women without radiographic knee OA was greater than that in men without radiographic knee OA [4]. In the present study, the prevalence of knee pain was 6–10 % in men with muscle strength ≥40 kgf, and 15–16 % in women with muscle strength ≥40 kgf. This high prevalence of knee pain in women with muscle strength ≥40 kgf, which is the reference point, may partly explain the lower OR for knee pain in women than men. The threshold of muscle strength for knee pain was similar or higher in women than men, which may indicate that factors associated with knee pain include not only gender but also weaker muscle strength.

There are limitations to the present study. This was a large-scale, population-based, cross-sectional study of baseline data. Thus, causal relationships could not be determined. For example, subjects with knee pain may have less physical activity, thereby leading to muscle atrophy and decreases in strength. Or, those individuals with knee pain may be less likely to perform to maximum capacity on the quadriceps strength assessment. The ROAD study is a longitudinal survey, so further progress may help elucidate any causal relationships. In addition, knee pain due to knee OA may not be rest pain, but mainly motion pain, and we did not classify pain into motion pain and rest pain. Therefore, pain in the present study may include not only that from knee OA but also that from other knee pathology.

## Conclusion

In conclusion, the present cross-sectional study using a large population from the ROAD study showed that quadriceps muscle strength rather than grip strength or muscle mass at the lower limbs was associated with knee pain. After adjustment for knee OA, muscle strength was independently associated with knee pain. The threshold of muscle strength for knee pain was similar in men and women. Further studies, along with continued longitudinal surveys in the ROAD study, will help improve our understanding of the relationship between muscle strength and pain.

## Additional file

Additional file 1: Figure S1. Quadriceps muscle strength by age strata (PPT 188 kb)
