# Supplementary material for: Quadriceps muscle strength, radiographic knee osteoarthritis and knee pain: the ROAD study
Source: BMC Musculoskelet Disord. 2015 Oct 16;16:305. doi: 10.1186/s12891-015-0737-5 (PMC4609096; doi:10.1186/s12891-015-0737-5)
Supplement: Additional file 1: Figure S1. — Quadriceps muscle strength by age strata (PPT 188 kb) [file 12891_2015_737_MOESM1_ESM.ppt]

## Slide 1
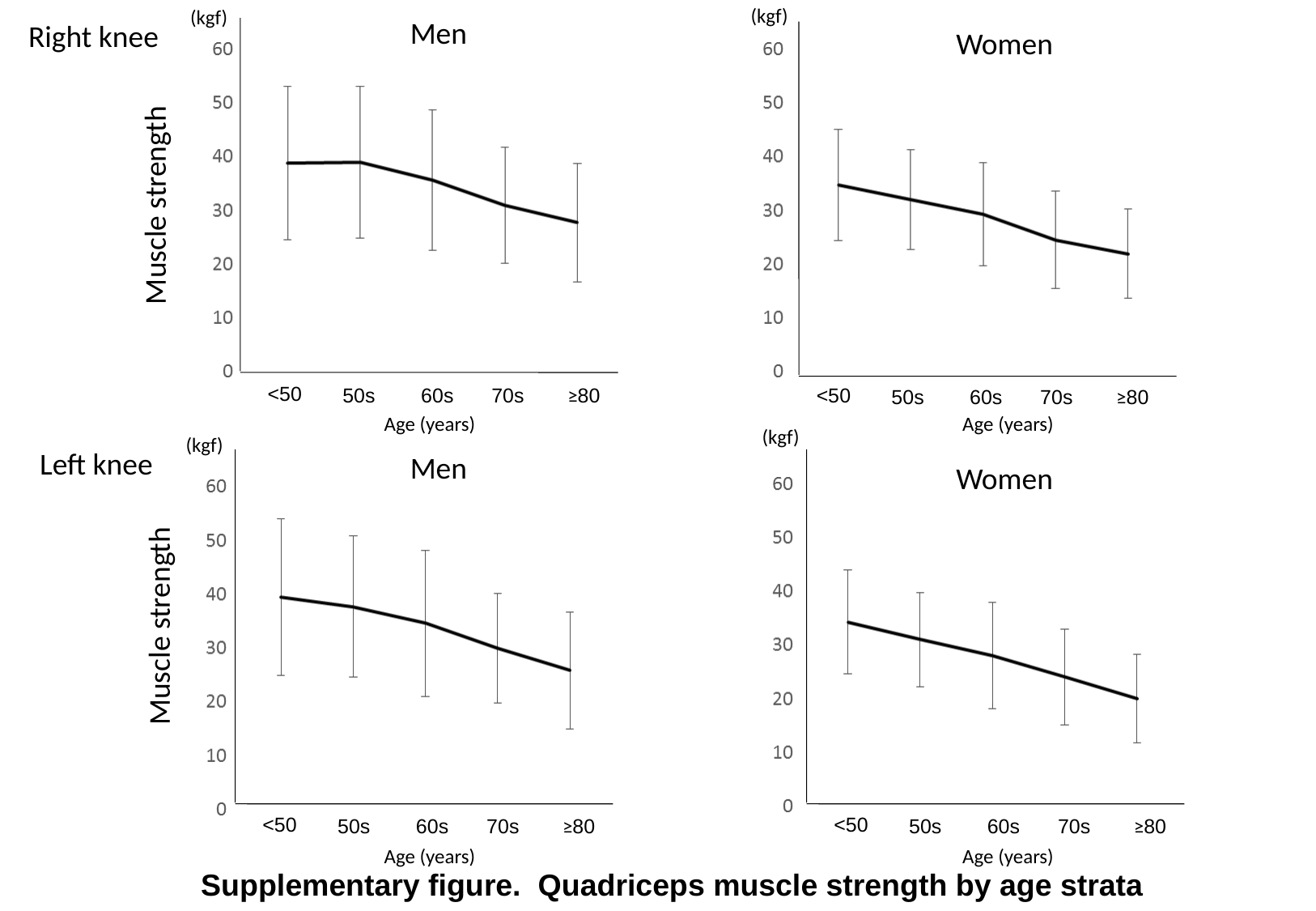

(kgf)
(kgf)
Men
Right knee
Women
Muscle strength
<50
50s
60s
70s
≥80
<50
50s
60s
70s
≥80
Age (years)
Age (years)
(kgf)
(kgf)
Left knee
Men
Women
Muscle strength
<50
<50
50s
60s
70s
≥80
50s
60s
70s
≥80
Age (years)
Age (years)
Supplementary figure. Quadriceps muscle strength by age strata
